# Field evaluation of a novel semi-quantitative point-of-care diagnostic for G6PD deficiency in Indonesia

**DOI:** 10.1371/journal.pone.0301506

**Published:** 2024-04-30

**Authors:** Arkasha Sadhewa, Lydia V. Panggalo, Illene Nanine, Ric N. Price, Kamala Thriemer, Ari W. Satyagraha, Benedikt Ley

**Affiliations:** 1 Global and Tropical Health Division, Menzies School of Health Research and Charles Darwin University, Darwin, Northern Territory, Australia; 2 EXEINS Health Initiative, Jakarta, Indonesia; 3 Mahidol-Oxford Tropical Medicine Research Unit (MORU), Faculty of Tropical Medicine, Mahidol University, Bangkok, Thailand; 4 Centre for Tropical Medicine and Global Health, Nuffield Department of Clinical Medicine, University of Oxford, Oxford, United Kingdom; 5 Eijkman Research Center for Molecular Biology, National Research and Innovation Agency, Cibinong, Indonesia; 6 Division of Education, Menzies School of Health Research and Charles Darwin University, Darwin, Northern Territory, Australia; Shoklo Malaria Research Unit, THAILAND

## Abstract

**Background:**

The WHO recommends routine testing of G6PD activity to guide radical cure in patients with *Plasmodium vivax* malaria. Females may have intermediate G6PD enzyme activity and to date, only complex diagnostics are able to reliably identify them. The semi-quantitative G6PD diagnostic “One Step G6PD Test” (Humasis, RoK; “RDT”) is a lateral flow assay that can distinguish deficient, intermediate, and normal G6PD status and offers a simpler diagnostic alternative.

**Methods:**

G6PD status of participants enrolled in Malinau and Nunukan Regencies and the capital Jakarta was assessed with the RDT, and G6PD activity was measured in duplicate by reference spectrophotometry. The adjusted male median (AMM) of the spectrophotometry measurements was defined as 100% activity; 70% and 30% of the AMM were defined as thresholds for intermediate and deficient G6PD status, respectively. Results were compared to those derived from spectrophotometry at the clinically relevant G6PD activity thresholds of 30% and 70%.

**Results:**

Of the 161 participants enrolled, 10 (6.2%) were G6PD deficient and 12 (7.5%) had intermediate G6PD activity by spectrophotometry. At the 30% threshold, the sensitivity of the RDT was 10.0% (95%CI: 0.3–44.5%) with a specificity of 99.3% (95%CI: 96.4–100.0%); the positive predictive value was 50.0% (95%CI: 1.3–98.7%) and the negative predictive value 94.3% (95%CI: 89.5–97.4%). The corresponding figures at the 70% threshold were 22.7% (95%CI: 7.8–45.4%), 100.0% (95%CI: 97.4–100.0%), 100.0% (95%CI: 47.8–100.0%) and 89.1% (95%CI: 83.1–93.5%), respectively.

**Conclusion:**

While there is a dire need for an easy-to-use, economical, semi-quantitative diagnostic for the point of care, the observed performance of the “One Step G6PD Test” in its current form was insufficient to guide antimalarial treatment.

## Introduction

Annually, between 4.9 to 14.3 million clinical cases of malaria are attributable to *Plasmodium vivax* (*P*. *vivax*), the second most prevalent malaria species globally [1, 2]. Vivax malaria causes significant health and economic burden largely as a consequence of the parasite’s ability to form dormant liver-stage parasites (hypnozoites) which can reactivate weeks to months after the initial infection, causing febrile relapses [3, 4]. The dormant hypnozoites represent a silent reservoir for malaria transmission that poses a significant barrier to malaria elimination and eradication.

The 8-aminoquinolines (8-AQ) class of antimalarial compounds are the only drugs that kill hypnozoites. Two 8-AQ drugs are currently licensed for vivax malaria treatment regimens: primaquine (PQ) and tafenoquine (TQ) [5, 6], both of which are co-administered with schizontocidal drugs to kill all infecting parasite stages of *P*. *vivax*; this treatment regimen is known as “radical cure” [7]. Although 8-AQ drugs are well-tolerated by most patients they are strong oxidants and can cause acute haemolysis in individuals with the common enzymopathy glucose-6-phosphate dehydrogenase (G6PD) deficiency [8].

G6PD deficiency (G6PDd) affects 400 to 500 million people globally [9], with the majority of those affected residing in currently or historically malaria-endemic countries [10]. G6PDd is caused by mutations in the G6PD gene, located on the X-chromosome, that impair the enzymes’ stability or catalytic activity, and thus the ability to maintain and regulate the redox potential in G6PD deficient red blood cells (RBC), rendering affected cells more vulnerable to oxidative stressors [11]. Since G6PDd is inherited in an X-linked manner, males are either hemizygous G6PD deficient (low enzyme activity) or hemizygous normal (normal enzyme activity). However, females can be either homozygous G6PD deficient (low enzyme activity), homozygous G6PD normal (normal enzyme activity), or heterozygous for the G6PD allele. Depending on lyonization pattern, the phenotypic G6PD activity of heterozygous females ranges from almost G6PD normal to deficient, with the majority clustering at around 50% activity [12, 13].

The World Health Organization (WHO) recommends that all patients are tested for G6PD status prior to treatment with an 8-AQ compound to avoid exposure in patients vulnerable to haemolysis [7, 14]. Although G6PD deficient patients can be diagnosed using fairly economical qualitative lateral flow diagnostics, which are easy to use, suitable for low-resource settings, and have rapid turnaround times [15], these tests cannot identify heterozygous females with intermediate G6PD activity, cannot adjust results for haemoglobin (Hb) levels, and result interpretation may be subjective [16]. Currently heterozygous females can only reliably be diagnosed using relatively expensive laboratory-based testing or more complex semi-quantitative point-of-care testing such as the STANDARD G6PD Test (SD Biosensor, RoK) [17]. In Indonesia, even with the availability of point-of-care testing, G6PD testing is not mandatory for routine care but only required in the event of relapse for the purposes of prescribing a higher dose of PQ [18].

A novel semi-quantitative lateral flow rapid diagnostic test (RDT), the One Step G6PD Test by Humasis (RoK, Cat No. AG6PD-7025), has recently been introduced to the market. The RDT claims to discriminate normal, intermediate, and deficient G6PD levels, and thus has the potential to be an affordable way of diagnosing patients with intermediate activity [19]. The RDT has been used in two surveys published in 2023 [20, 21], though its performance is unknown. The aim of this study was to assess the performance of the Humasis One Step G6PD Test in field settings and compare its results with that of reference method spectrophotometry.

## Methods

### Overview

**Fig 1:**
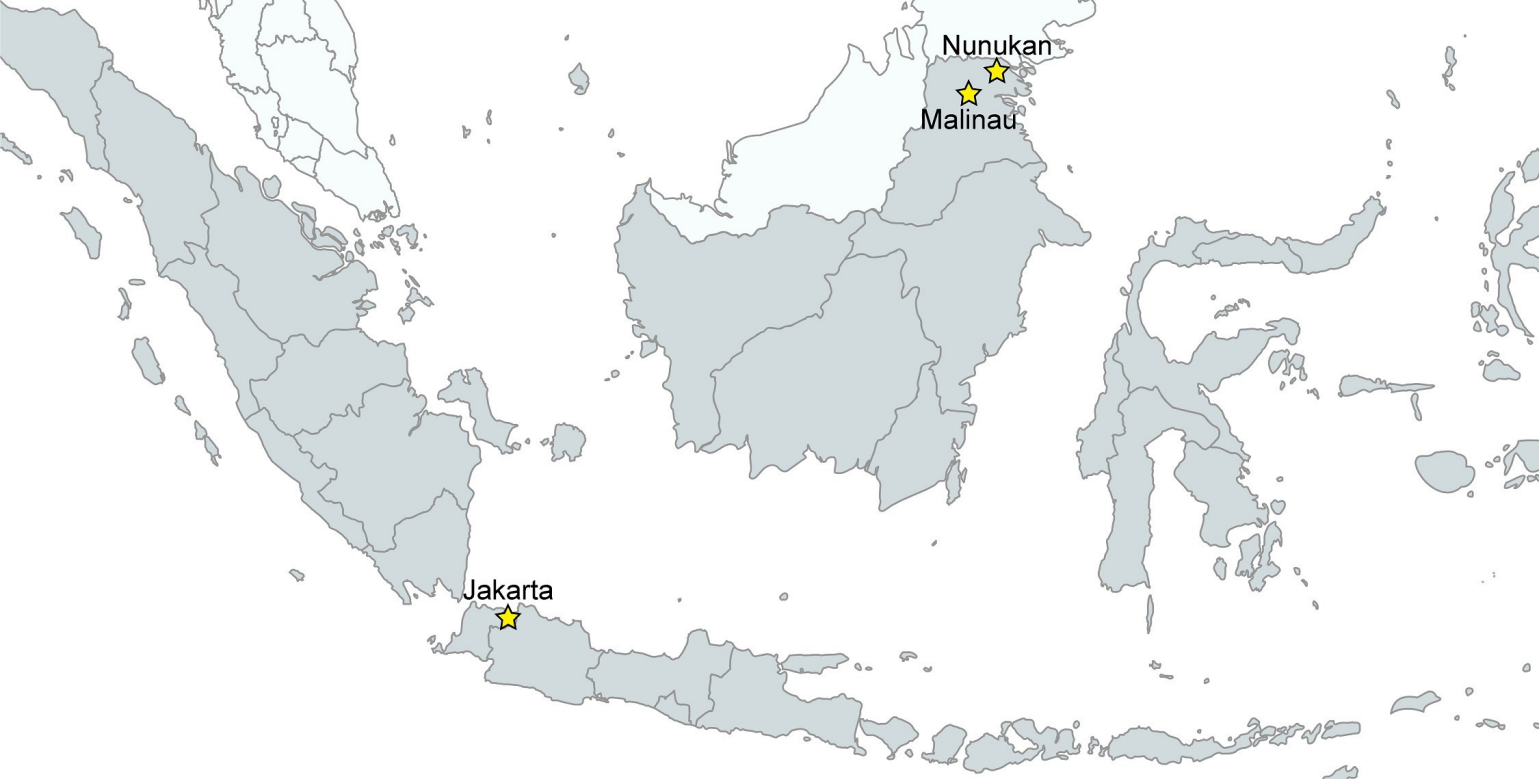
Map pointing of the study sites created in the MapChart website [22] and modified accordingly.

**Fig 2:**
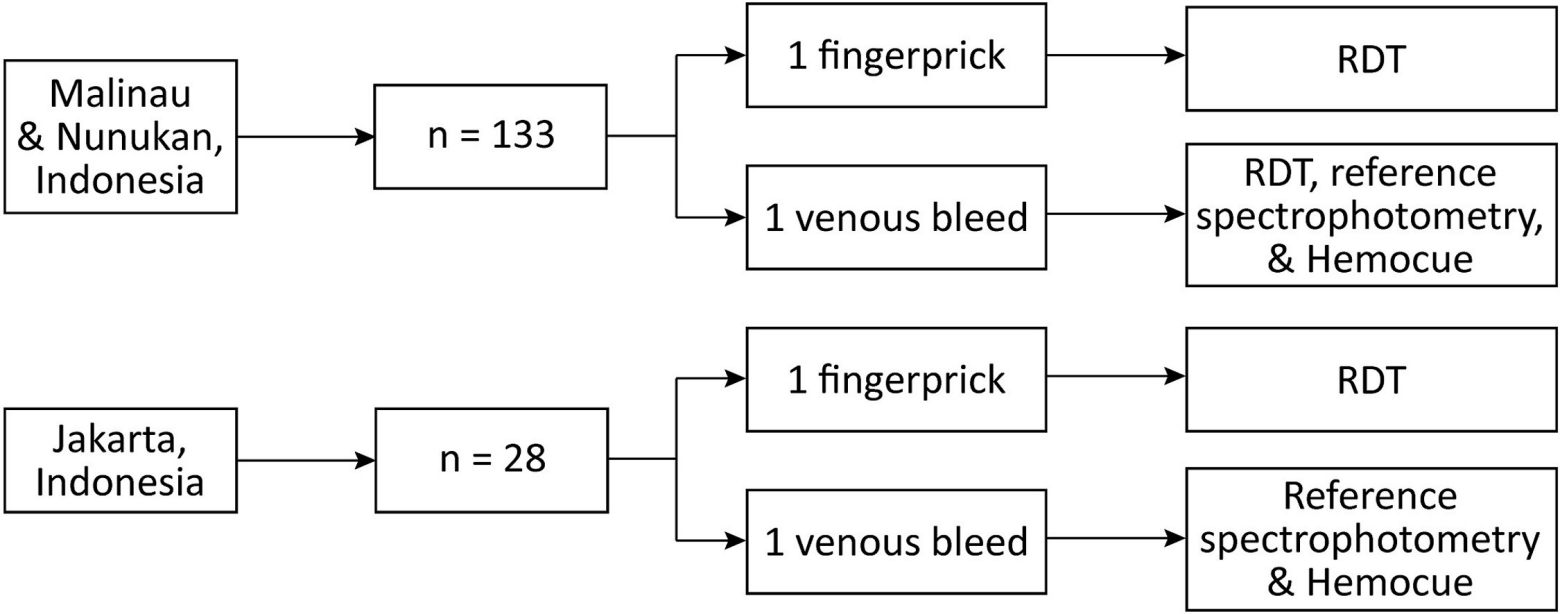
Schematic workflow of the field studies in Malinau and Nunukan Regencies, and Jakarta, Indonesia.

Study participants were enrolled at three sites in Indonesia: Malinau Regency and Nunukan Regency, both in North Kalimantan Province, and in the capital Jakarta (Fig 1). All individuals aged six years or older visiting Pulau Sapi (Malinau) and Sanur (Nunukan) community health centers (Puskesmas) were invited to participate. In Jakarta, individuals aged six years or older who self-reported as G6PD deficient based on qualitative, quantitative, genotyping, or any other clinical diagnosis were purposefully enrolled. Family members of participants identified in Jakarta were also invited to participate to include as many individuals with low G6PD activity as possible. Written informed consent was collected from all participants or their legal guardians prior to enrolment (Fig 2).

### Ethics

Ethics approvals were obtained from the Human Research Ethics Committee (HREC) of the Northern Territory Health, Australia (Menzies HREC 22–4346) and the Atma Jaya Catholic University Research Ethics Committee, Indonesia (No. 0008A/III/PPPE.PM.10.05/09/2022) before the first participant was enrolled.

### Blood collection and storage

Capillary blood from a finger prick was collected from all participants for RDT testing, and 3 mL of venous blood for reference testing by spectrophotometry were collected and stored in K2 EDTA vacutainer tubes (BD, USA; 3 mL). Venous blood samples were stored at 4°C between blood collection and testing. RDT Testing with capillary blood and venous blood was performed immediately after blood collection at the Puskesmas Pulau Sapi in Malinau, Puskesmas Sanur in Nunukan, and the laboratory facility at Exeins Health Initiative in Jakarta. Reference testing was performed in a laboratory in Malinau City on the day of blood collection for Malinau participants, and less than 48 hours after blood collection for Nunukan participants. In Jakarta reference testing was done on the same day as blood collection at the laboratory of the Exeins Health Initiative.

### RDT testing

**Fig 3:**
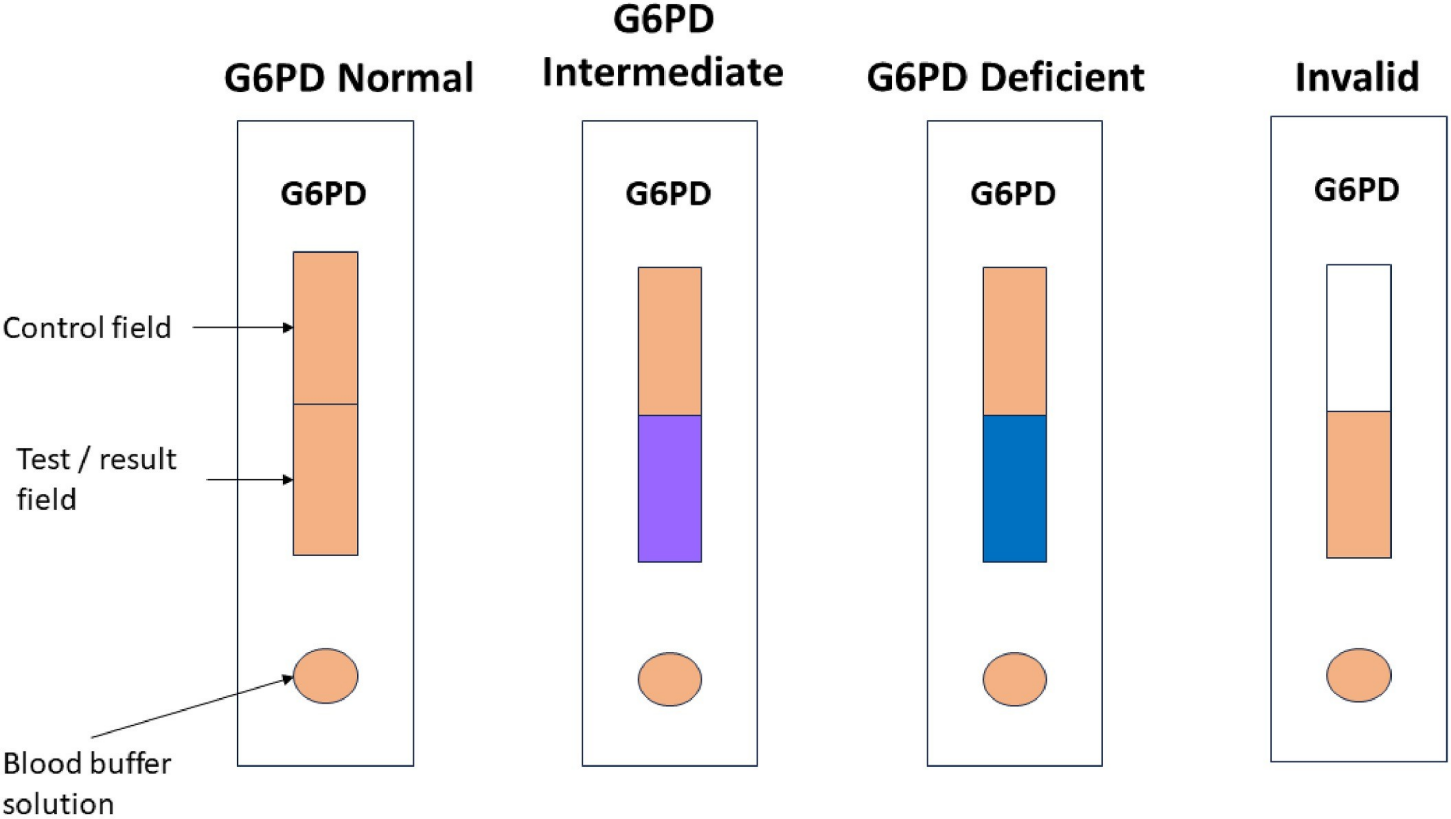
Test interpretation of the One-Step G6PD test.

The Humasis One-Step G6PD test (“RDT”) is a semi-quantitative colorimetric lateral flow assay that categorizes G6PD activities as deficient, intermediate, or normal within five minutes. The assay is provided in cassette format with a test membrane. A red color change of the test membrane indicates normal G6PD activity, purple indicates intermediate activity, and blue indicates deficient activity (Fig 3).

All RDTs used were from the same lot. Following manufacturer recommendations 5 μL of fresh capillary or venous blood were added to a lysis buffer using a micro-dropper (buffer and dropper are included in the test kit) and thoroughly mixed by pipetting up and down with the micro-dropper. The buffer tube was closed with a lid that included a dropper, and two drops of the blood-buffer solution were dispensed to the sample well of the lateral flow assay directly from the tube’s dropper lid. Any color change of the test membrane was recorded after five minutes and interpreted following the test’s instructions for use (Fig 3).

In the participants from Jakarta, only capillary blood was tested for G6PD deficiency using the RDT, while in participants from Malinau and Nunukan Regencies, venous blood stored in EDTA tubes was also tested. In all cases the RDT was read by two independent technicians who had completed standardized training and were blinded towards the other result. In case of contradictory findings, the test was repeated from the same sample (no additional sampling was done). The same technicians repeated testing on the venous samples (no venous testing was done in Jakarta) and were therefore aware of the RDT result from capillary testing. In Jakarta technicians were aware that only participants that claimed to have low G6PD activities were enrolled.

### Spectrophotometry (Reference method)

G6PD activity was measured from venous blood samples by spectrophotometry using a commercial kit (Pointe Scientific, USA) at 340 nm wavelength on a machine from Biochrom WPA (Biowave II UV/Vis, UK) at 37°C temperature, following instructions from the kit’s manufacturer [23]. Quality assurance of the spectrophotometry readings was conducted each day using G6PD deficient, intermediate, and normal controls (ACS Analytics, USA; Cat. Nos. HC-108DE, HC-108IN, and HC-108, respectively). Hb readings from a Hb 301 device (Hemocue, USA) done at the same time as reference testing were used to normalize G6PD activity. G6PD activity measurement by spectrophotometry was done in duplicate for each sample. When the difference between duplicate measurements was larger than 1.0 U/g Hb, the duplicate measurements were repeated. Average G6PD activity from the duplicate readings was used to categorize the participants’ G6PD status. The same technicians that did RDT testing also did reference testing by spectrophotometry, technicians were accordingly not blinded towards the RDT result.

### Genotyping

Samples from male participants with less than 30% G6PD activity and females with less than 70% activity, and samples from all participants purposively enrolled in Jakarta were genotyped for the underlying G6PD variant. DNA was extracted from whole venous blood stored in EDTA tubes (BD, USA) using a commercial kit (QIAmp DNA Blood Mini Kit, Qiagen, Cat No. 51104). Variant-specific genotyping was performed by PCR-RFLP for common variants found in Indonesia following previously published PCR and RFLP protocols (Vanua Lava [24], Viangchan [25], Mahidol and Kaiping [26], Mediterranean [27], Coimbra [28], and Chatham [29]).

### Statistical analysis and sample size calculation

Data were recorded on case report forms (paper-based) and digitalized with EpiData version 3.1 (EpiData Association, Denmark). Data organization and analysis were done in STATA versions 13, 15, and 17 (Stata Corp, USA) [30].

The adjusted male median (AMM) was calculated from the spectrophotometry readings of male participants of the Malinau and Nunukan Regencies sites and defined as 100% G6PD activity. Following the calculation described by Domingo et al [31], the adjusted male median was the median calculated from all male participants having G6PD activity >10% of the study population’s male median. Any participant with less than 30% activity was categorized as G6PD deficient, participants with activities between 30% and less than 70% activity were categorized as intermediate, and all others were considered G6PD normal. The sensitivity and specificity of the RDT were calculated using standard formulae considering spectrophotometry as reference method [32]. To calculate the performance of the RDT in diagnosing deficient individuals, a true positive result was defined as a sample having a deficient result by RDT and G6PD activity of ≤30% by spectrophotometry. To calculate the performance for intermediate RDT results, any sample that triggered a deficient or intermediate RDT result and had ≤70% activity by spectrophotometry was defined as a true positive result [32].

To determine sensitivity and specificity at the 30% cut-off of at least 90% with 18% absolute confidence interval, assuming procedural errors in 10% of all cases and a G6PD deficiency (<30% of normal activity) prevalence of 7.5% [10], required a total sample size of 162 participants [33].

## Results

**Fig 4:**
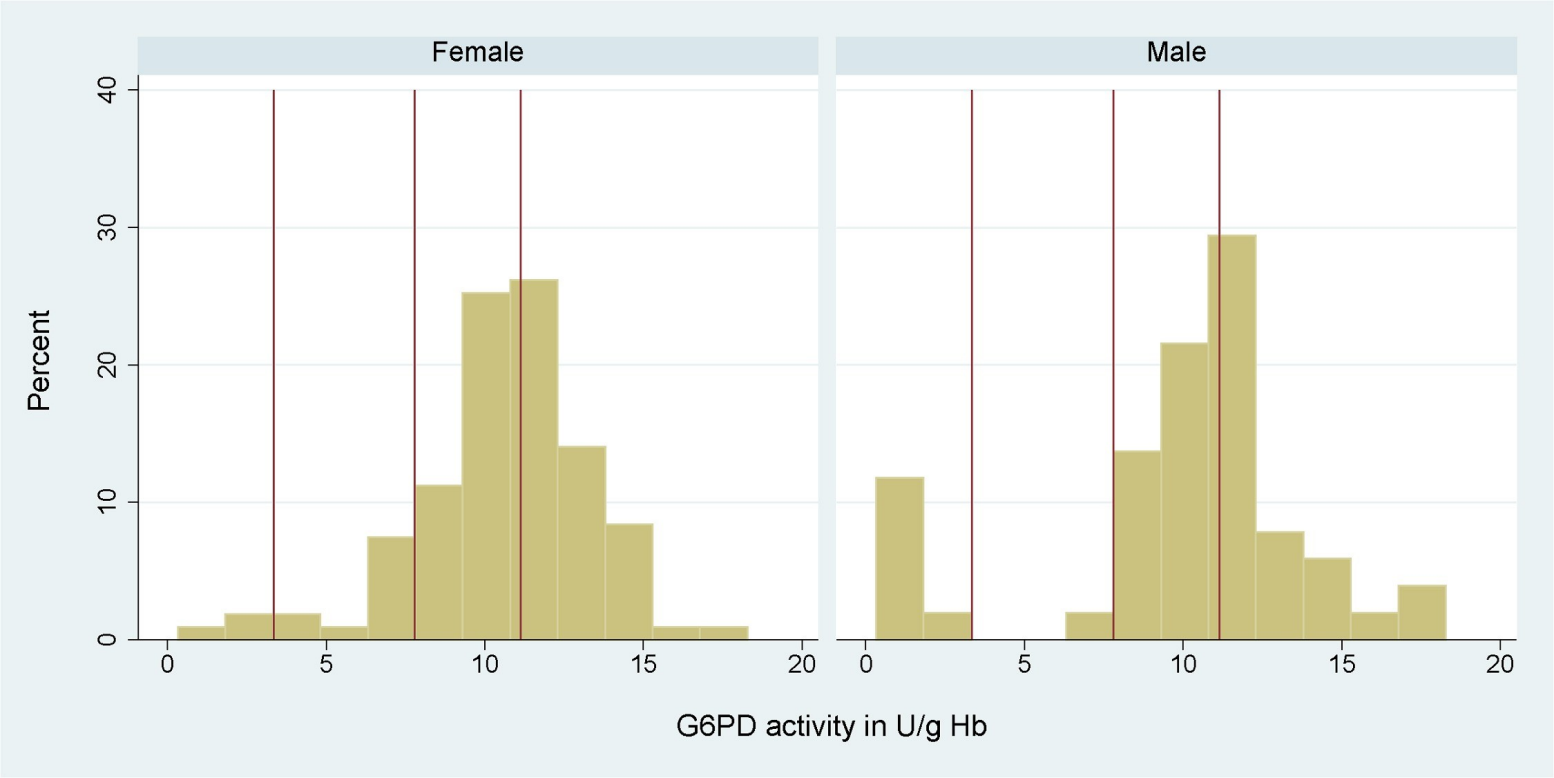
Histogram of G6PD activity as measured by spectrophotometry of female and male participants from Indonesia (n = 158). Vertical red lines denote (from left to right) 30%, 70% and 100% of AMM. Sex was not recorded in 3 participants.

In total 161 participants were enrolled in this study (S1 Data). 133 individuals were enrolled in Malinau and Nunukan Regencies between the 24th and 28th of November 2022 and an additional 28 individuals (all thought to be G6PD deficient or intermediate) were enrolled in Jakarta between the 6th of January and 7th of July 2023. The AMM was calculated to be 11.1 U/g Hb (IQR: 9.1 to 12.2) based on spectrophotometry and defined as 100% G6PD activity (Fig 4 and Table 1).

**Table 1:** Demography and G6PD activity of the study populations.

Site	Malinau and Nunukan	Jakarta
Male	41	10
Female	89	18
Unknown	3	0
Total	133	28
Median age in years (range)	36.5 (29 to 47)^§^	37 (23 to 56)†
Median Hb in g/dL (IQR)	13.7 (12.7 to 14.5)	14.1 (12.9 to 15.0)
AMM (IQR)*	11.1 (9.1 to 12.2)	NA
**G6PD Deficient (Activity <30% AMM)**		
Male (%)	0	7
Female (%)	1	2
**G6PD Intermediate (Activity 30–70% AMM)**		
Male (%)	1	0
Female (%)	2	9
**G6PD Normal (Activity >70% AMM)**		
Male (%)	40	3
Female (%)	86	7
Unknown	3	NA

^§^Age data from 100/133 participants.

†Age data from 25/28 participants.

*AMM was calculated from the male participants in the Malinau and Nunukan Regencies study population (n = 41) by spectrophotometry. When males from the Jakarta study population were included, the AMM was less than 3% lower.

Based on spectrophotometry, out of the 133 participants enrolled in Malinau and Nunukan Regencies, 1 (0.8%) participant was G6PD deficient and 3 (2.3%) had intermediate G6PD activities. In Jakarta 18 out of the 28 purposively enrolled participants had G6PD activities by spectrophotometry below 70%, 9 individuals were G6PD deficient, 9 individuals were categorized as intermediate, while 10 participants had G6PD normal activities (Table 1). One of the participants with intermediate G6PD activity was male and not genotyped. 21 samples were genotyped and 3 different G6PD variants in 12 samples were identified, confirming the spectrophotometry results (Table 2). All participants were informed of the results from spectrophotometry and genotyping.

**Table 2:** Summary of genotyping results of male participants with deficient G6PD activity and female participants with deficient and intermediate G6PD activity.

G6PD category	N	Viangchan	Chatham	Kaiping	No variant identified*
Deficient	10	1 Hetero. 2 Homo. 2 Hemi.	1 Hemi.	2 Hemi.	2
Intermediate	11	2 Hetero.	2 Hetero.	0	7

Hetero = heterozygous, Homo = Homozygous, Hemi = Hemizygous

*No variant found among the variant-specific PCR-RFLP employed in this study

The RDT was read by two independent technicians and their results matched in all cases. The RDT correctly classified all G6PD normal individuals (n = 139), but only identified 10.0% (1/10) of participants with less than 30% activity and 8.3% (1/12) of participants with activity between 30–70% (S1 Table). In Malinau and Nunukan Regencies, both capillary and venous blood samples were tested with the RDT and returned identical results (S2 Table). No invalid results were recorded at any site.

Observed sensitivity at the 30% activity threshold was 10.0% (95%CI: 0.3 to 44.5%) and specificity was 99.3% (95%CI: 96.4 to 100.0%). The corresponding sensitivity for an intermediate RDT result (70% activity threshold) was 22.7% (95%CI: 7.8 to 45.4%) and 100.0% (95%CI: 97.4 to 100.0%; Table 3 and S1 Fig).

**Table 3:** Performance of the RDT against reference spectrophotometry.

Reference spectrophotometry cut-off	Prevalence%	Sensitivity% (95%CI) (TP/(TP+FN)	Specificity% (95%CI) (TN/(TN+FP)	PPV% (95%CI) (TP/(TP+FP)	NPV% (95%CI) (TN/(TN+FN)
All intermediate results by RDT considered as G6PD normal					
30% AMM	6.2%	**10.0% (0.3–44.5%) (1/10)**	**99.3% (96.4–100.0%) (150/151)**	**50.0% (1.3–98.7%) (1/2)**	**94.3% (89.5–97.4%) (150/159)**
70% AMM	13.7%	*9.1% (1.1–29.2%) (2/22)*	*100.0% (97.4–100.0%) (139/139)*	*100.0% (15.8–100.0%) (2/2)*	*87.4% (81.2–92.1%) (139/159)*
All intermediate results by RDT considered as G6PD deficient					
30% AMM	6.2%	*30.0% (6.7–65.2%) (3/10)*	*98.7% (95.3–99.8%) (149/151)*	*60.0% (14.7–94.7%) (3/5)*	*95.5% (91.0–98.2%) (149/156)*
70% AMM	13.7%	**22.7% (7.8–45.4%) (5/22)**	**100.0% (97.4–100.0%) (139/139)**	**100.0% (47.8–100.0%) (5/5)**	**89.1% (83.1–93.5%) (139/156)**

When projecting varying prevalence of G6PD deficiency from 1% to 20% to the observed sensitivity and specificity (Table 4), the positive predictive value (PPV) increased from 13.2% (95%CI: 1.0 to 69.3%) at 1% prevalence to 79.1% (95%CI: 20.3 to 98.2%) at 20% prevalence, while the negative predictive value (NPV) decreased from 99.1% (95%CI: 98.9 to 99.3%; 1% prevalence) to 81.5% (95%CI: 78.2 to 84.4%; 20% prevalence). When considering the performance to diagnose intermediate and deficient individuals the PPV remained consistent at 100% and the NPV decreased from 99.2% (95%CI: 99.0 to 99.4%) to 83.8% (95%CI: 80.5 to 86.7%) (Table 4).

**Table 4:** PPV and NPV for different G6PD deficiency prevalence based on the performance of the RDT in identifying deficient and deficient / intermediate individuals (Table 3).

Prevalence	Sensitivity	Specificity	PPV (95%CI)	NPV (95%CI)
Predictive values to identify deficient individuals (<30% activity)				
1.0%	10.0%	99.3%	13.2% (1.0–69.3%)	99.1% (98.9–99.3%)
3.0%	10.0%	99.3%	31.8% (3.1–87.4%)	97.3% (96.7–97.8%)
5.0%	10.0%	99.3%	44.3% (5.1–92.2%)	95.4% (94.5–96.3%)
8.0%	10.0%	99.3%	56.8% (8.1–95.1%)	92.7% (91.2–94.0%)
10.0%	10.0%	99.3%	62.7% (10.2–96.1%)	90.0% (89.0–92.4%)
20.0%	10.0%	99.3%	79.1% (20.3–98.2%)	81.5% (78.2–84.4%)
Predictive values to identify deficient/intermediate individuals (<70% activity)				
1.0%	22.7%	100.0%	100% (NA)	99.2% (99.0–99.4%)
3.0%	22.7%	100.0%	100% (NA)	97.7% (97.1–98.1%)
5.0%	22.7%	100.0%	100% (NA)	96.1% (95.1–96.9%)
8.0%	22.7%	100.0%	100% (NA)	93.7% (92.2–94.9%)
10.0%	22.7%	100.0%	100% (NA)	92.1% (90.3–93.6%)
20.0%	22.7%	100.0%	100% (NA)	83.8% (80.5–86.7%)

## Discussion

This is the first field evaluation of the novel G6PD semi-quantitative lateral flow assay manufactured by Humasis. The observed sensitivity was ≤30% across all clinically relevant G6PD activity thresholds, irrespective of whether intermediate results by RDT were categorized as deficient or G6PD normal. The clinical application of G6PD testing is to guide the administration of radical cure, and in this scenario the negative predictive value (NPV) is more relevant than sensitivity or specificity and is dependent on the underlying prevalence of G6PD deficiency. The NPV reflects the proportion of individuals that would erroneously receive treatment that can potentially trigger severe hemolysis if the diagnostic was considered. Based on the observed sensitivity and specificity, the NPV of the RDT at 8% prevalence (estimated global G6PDd prevalence [10]) was less than 93% at the 30% enzyme activity cut-off and less than 94% at the 70% cut-off. In other words, the proportion of false G6PD normal (false negative) results would be more than 7% for deficient individuals only and more than 6% for deficient and intermediate participants (Table 4). The WHO has defined target product profiles (TPP) for G6PD point of care diagnostics and recommends ≥95% positive agreement, ≥90% negative agreement at the 30% activity threshold and ≥85% positive agreement and ≥90% negative agreement at the 70% activity threshold [34]. While the negative agreement at the 30% activity cut-off (94.3%), and the positive agreement at the 70% cut-off (100.0%) observed in our study met the recommended WHO TPP criteria, the positive agreement of 50% at the 30% activity threshold and the negative agreement of 89.1% at the 70% activity threshold (Table 3) were well below the accepted WHO criteria [34].

The reaction time of the test according to manufacturer’s instructions is five minutes. In Jakarta only participants that claimed to be G6PD deficient or intermediate were enrolled, accordingly laboratory technicians could have been biased in their interpretation. Interestingly non-systematic observations during this evaluation suggested that the reaction time may be too long. Within five minutes even blood samples with very low G6PD activity, as later confirmed by spectrophotometry and genotyping (S3 Table), appeared to trigger a color change. It may therefore be possible that performance could be improved if the reaction time was shortened.

Procedures of the RDT include a pipetting step to lyse the blood sample, rendering procedures more complex than those of other lateral flow assays and comparable to procedures required for the most widely used semi-quantitative point of care diagnostic, the STANDARD G6PD Test by SD Biosensor (ROK) [35–37]. The cost of the RDT in Indonesia at time of publication were approximately 2.90 USD, lower than other commercially available G6PD point of care diagnostics that can identify individuals with intermediate G6PD activities [17] and in contrast to other diagnostics the RDT does not need further laboratory equipment or machinery. The recommended storage temperatures for the RDT are between 1–30°C, which may be challenging to maintain in remote tropical areas.

Over the last years a number of lateral flow G6PD assays have been evaluated in field trials, including the CareStart G6PD RDT from CareStart (RoK) and the BinaxNOW G6PD Test from Binax (USA) [38, 39], both of which return binary results and only detect G6PD deficiency at the 30% G6PD activity threshold [38, 39]. Whilst the semi-quantitative RDT offers potentially three diagnostic categories its performance was inferior; the CareStart test had a pooled sensitivity of 96% and specificity of 95% [15] and the corresponding values for the BinaxNOW test were 55–100% and 97–100% respectively [39–41].

To the knowledge of the authors two studies using the RDT have been published to date. One cross-sectional survey in Papua, Indonesia reported a G6PD deficiency prevalence as determined by the RDT of 21%, which is in stark contrast to other reports from the area that reported local phenotypic G6PD prevalence of 2.6% [20, 42, 43]. A second survey among 27 neonates with jaundice in Samarinda, Indonesia found an RDT-based G6PD deficiency prevalence of 59%; G6PD deficiency is a known risk factor for neonatal jaundice [21, 44].

Our findings must be considered with caution as the number of deficient and intermediate individuals enrolled was lower than expected, accordingly the variation around the calculated performance indicators was large and warrants additional analysis with a larger cohort of deficient and intermediate individuals. Participants were also enrolled at two distinct locations: 83% (133/161) in Malinau and Nunukan Regencies (both in North Kalimantan Province) and 17% (28/161) of participants were enrolled in Jakarta. The AMM for the reference spectrophotometry was calculated from Malinau and Nunukan Regencies male participants (n = 41) reflecting the purposive recruitment of the Jakarta participants. However, when recalculating the AMM including all male participants (n = 51), the revised AMM (10.8 U/g Hb) was less than 3% lower than the AMM that was used in this study (11.1 U/g Hb). Finally, the same two technicians performed RDT and reference testing. Both were blinded towards the other technicians’ findings and towards the G6PD status of participants in Malinau and Nunukan for the capillary samples. However, the second reading on venous samples as well as the subsequent reference testing by spectrophotometry was done by the same individuals and we cannot exclude that this may have impacted these readings. In Jakarta only capillary samples were tested, but technicians were aware that participants enrolled at that site all claimed to be G6PD deficient.

In conclusion the format and pricing of the RDT make it an economical point of care diagnostic, however the observed performance does not render the assay suitable for deployment at this stage.

## Supporting information

S1 Fig Dot plot of spectrophotometry results (in % AMM) against deficiency category as defined by the Humasis RDT (n = 161). Horizontal red lines indicate 30%, 70%, and 100% G6PD activity of the AMM.
(TIF)

S1 Table 2x2 table of the Humasis RDT (capillary blood) and reference spectrophotometry (venous blood) for all study participants (n = 161). (DOCX)

S2 Table 2x2 table of capillary vs venous blood, in Malinau and Nunukan Regencies study participants (n = 133). (DOCX)

S3 Table List of male participants with deficient G6PD activity and female participants with deficient or intermediate G6PD activity and the corresponding results of genotyping by PCR-RFLP. (DOCX)

S1 Data Corresponding database. (XLSX)
