# Supplementary figures and images for: Field evaluation of a novel semi-quantitative point-of-care diagnostic for G6PD deficiency in Indonesia
Source: PLoS One. 2024 Apr 30;19(4):e0301506. doi: 10.1371/journal.pone.0301506 (PMC11060553; doi:10.1371/journal.pone.0301506)

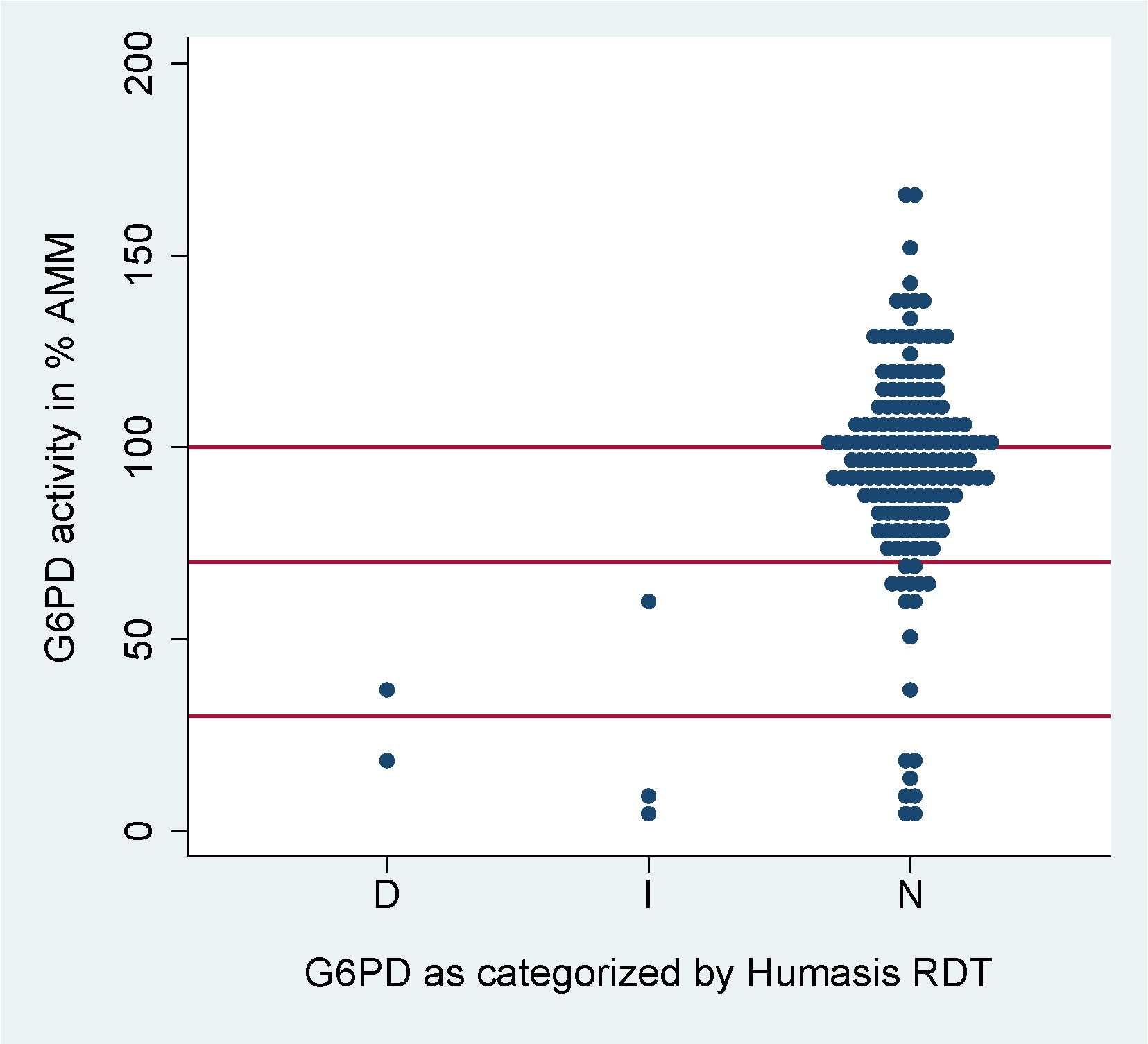

Supplement: S1 Fig — Horizontal red lines indicate 30%, 70%, and 100% G6PD activity of the AMM. (TIF) [file pone.0301506.s001.tif]
