# Supplementary material for: Field evaluation of a novel semi-quantitative point-of-care diagnostic for G6PD deficiency in Indonesia
Source: PLoS One. 2024 Apr 30;19(4):e0301506. doi: 10.1371/journal.pone.0301506 (PMC11060553; doi:10.1371/journal.pone.0301506)
Supplement: S1 Table — (DOCX) [file pone.0301506.s002.docx]

**Table S1.** 2x2 table of the Humasis RDT (capillary blood) and reference spectrophotometry (venous blood) for all study participants (n=161).

|  |  | Clinically relevant activity thresholds (% AMM) | | | |
| --- | --- | --- | --- | --- | --- |
|  |  | <30% | 30-70% | >70% | Total |
| Humasis | Deficient | 1 | 1 | 0 | 2 |
|  | Intermediate | 2 | 1 | 0 | 3 |
|  | Normal | 7 | 10 | 139 | 156 |
|  | Total | 10 | 12 | 139 | 161 |
