# Supplementary material for: Field evaluation of a novel semi-quantitative point-of-care diagnostic for G6PD deficiency in Indonesia
Source: PLoS One. 2024 Apr 30;19(4):e0301506. doi: 10.1371/journal.pone.0301506 (PMC11060553; doi:10.1371/journal.pone.0301506)
Supplement: S2 Table — (DOCX) [file pone.0301506.s003.docx]

**Table S2.** 2x2 table of capillary vs venous blood, in Malinau and Nunukan Regencies study participants (n=133).

|  |  | Capillary Blood | | |
| --- | --- | --- | --- | --- |
|  |  | Deficient | Intermediate | Normal |
| Venous Blood | Deficient | 0 | 0 | 0 |
|  | Intermediate | 0 | 0 | 0 |
|  | Normal | 0 | 0 | 133 |
