# Supplementary material for: Field evaluation of a novel semi-quantitative point-of-care diagnostic for G6PD deficiency in Indonesia
Source: PLoS One. 2024 Apr 30;19(4):e0301506. doi: 10.1371/journal.pone.0301506 (PMC11060553; doi:10.1371/journal.pone.0301506)
Supplement: S3 Table — (DOCX) [file pone.0301506.s004.docx]

**Table S3.** List of male participants with deficient G6PD activity and female participants with deficient or intermediate G6PD activity and the corresponding results of genotyping by PCR-RFLP

| **ID** | **Sex** | **G6PD Status by RDT** | **G6PD Activity by Spectrophotometry (U/g Hb)** | **G6PD Activity in % AMM** | **G6PD Status by Spectrophotometry** | **Genotype by PCR-RFLP*** |
| --- | --- | --- | --- | --- | --- | --- |
| MLU 015 | F | N | 2.1 | 19.1% | D | Viangchan hetero |
| MLU 060 | F | N | 7.1 | 63.9% | I | Not identified |
| MLU 156 | F | N | 7.8 | 69.6% | I | Not identified |
| MLU 181 | F | N | 4.1 | 36.9% | I | Viangchan hetero |
| MLU 183 | F | N | 0.8 | 7.4% | D | Viangchan homo |
| MLU 184 | M | N | 1.9 | 17.3% | D | Viangchan hemi |
| MLU 187 | M | I | 1.2 | 10.7% | D | Kaiping hemi |
| MLU 188 | F | D | 2.1 | 18.5% | D | Viangchan homo |
| MLU 189 | F | D | 3.9 | 35.1% | I | Not identified |
| MLU 190 | F | N | 7.1 | 63.3% | I | Chatam hetero |
| MLU 192 | F | N | 7.0 | 62.9% | I | Chatam hetero |
| MLU 193 | F | N | 5.7 | 51.2% | I | Not identified |
| MLU 194 | M | I | 0.6 | 5.8% | D | Not identified |
| MLU 195 | M | N | 0.8 | 6.9% | D | Chatam hemi |
| MLU 196 | F | I | 6.8 | 60.7% | I | Not identified |
| MLU 199 | M | N | 1.6 | 14.2% | D | Kaiping hemi |
| MLU 200 | F | N | 7.0 | 63.0% | I | Viangchan hetero |
| MLU 205 | M | N | 0.9 | 7.7% | D | Viangchan hemi |
| MLU 207 | F | N | 7.3 | 65.8% | I | Not identified |
| MLU 208 | M | N | 0.3 | 3.0% | D | Not identified |
| MLU 209 | F | N | 6.7 | 59.9% | I | Not identified |

*Hetero = heterozygous, Homo = Homozygous, Hemi = Hemizygous*

*M = male, F = female*

*N = normal, I = intermediate, D = deficient*

**Variant-specific genotyping for variants Chatham, Coimbra, Kaiping, Mahidol, Mediterranean, Vanua Lava, and Viangchan*
